# Proteinuria Increases the PLASMIC and French Scores Performance to Predict Thrombotic Thrombocytopenic Purpura in Patients With Thrombotic Microangiopathy Syndrome

**DOI:** 10.1016/j.ekir.2021.11.009

**Published:** 2021-11-16

**Authors:** Nicolas Fage, Corentin Orvain, Nicolas Henry, Chloé Mellaza, François Beloncle, Marie Tuffigo, Franck Geneviève, Paul Coppo, Jean François Augusto, Benoit Brilland

**Affiliations:** 1 Service de Néphrologie-Dialyse-Transplantation, Université d’Angers, Centre Hospitalier Universitaire Angers, Angers, France; 2 Service de Médecine Intensive Réanimation, Médecine Hyperbare, Centre Hospitalier Universitaire Angers, Angers, France; 3 Service des Maladies du sang, Centre Hospitalier Universitaire d’Angers, Angers, France; 4 Service de Néphrologie et hémodialyse, CH Laval, Laval, France; 5 Laboratoire d’Hématologie, Université d’Angers, Centre Hospitalier Universitaire Angers, Angers, France; 6 Centre de Référence des Microangiopathies Thrombotiques, Hôpital Saint-Antoine, Assistance Publique—HóPitaux De Paris, Paris, France; 7 Université d’Angers, Université de Nantes, CHU d’Angers, Inserm, CRCINA, SFR ICAT, F-49000 Angers, France

**Keywords:** French score, PLASMIC score, proteinuria, thrombotic microangiopathy syndrome, thrombotic thrombocytopenic purpura

## Abstract

**Introduction:**

PLASMIC and French scores have been developed to help clinicians in the early identification of patients with thrombotic thrombocytopenic purpura (TTP). Nevertheless, the validity of these scores in thrombotic microangiopathy (TMA) cohorts with low TTP prevalence remains uncertain. We aimed to evaluate their diagnostic value in routine clinical practice using an unselected cohort of patients with TMA. We also analyzed the value of adding proteinuria level to the scores.

**Methods:**

We retrospectively included all patients presenting with a biological TMA syndrome between January 1, 2008, and December 31, 2019, in a tertiary hospital. TMA etiology was ascertained, and scores were evaluated. Modified scores, built by adding 1 point for low proteinuria (<1.2 g/g), were compared with original scores for TTP prediction.

**Results:**

Among 273 patients presenting with a full biological TMA syndrome, 238 were classified with a TMA diagnosis. Complete scores and proteinuria level were available in 134 patients with a TTP prevalence of 7.5%. Area under the receiver operating characteristic curve (AUC) of PLASMIC and French scores for TTP diagnosis was 0.65 (0.46–0.84) and 0.72 (0.51–0.93), respectively. AUC of modified PLASMIC and French scores was 0.76 (0.59–0.92) (*P* = 0.003 vs. standard score) and 0.81 (0.67–0.95) (*P* = 0.069 vs. standard score), respectively. Specificity, positive predictive value (PPV), and positive likelihood ratio of high-risk scores were significantly improved by adding proteinuria level.

**Conclusion:**

PLASMIC and French scores have low predictive values when applied to an unselected TMA cohort. Including proteinuria level in the original scores improves their performance for TTP prediction.

## Graphical abstract

**Figure:**
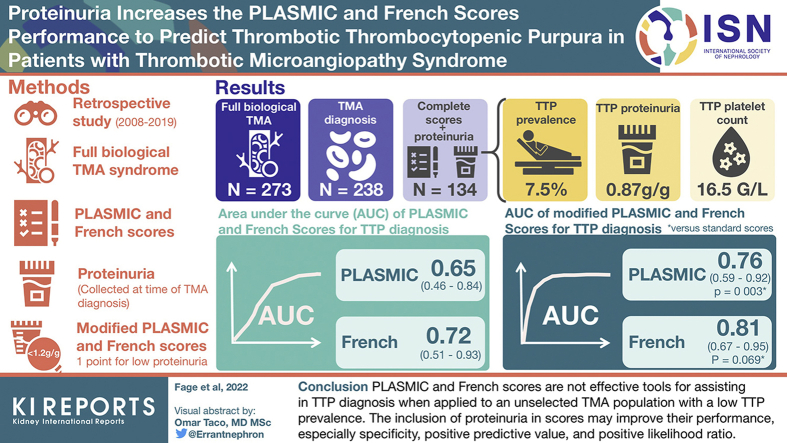

TMA is characterized by hemolytic anemia, thrombocytopenia, and ischemic organ injury.^1^ It can be classified as primary (TTP and atypical hemolytic and uremic syndrome [a-HUS]) and secondary (typical HUS; pregnancy-, drug-, infection-, active malignancy-, malignant hypertension-, transplantation-, and autoimmune disease-associated TMA). The pathophysiology of TTP is driven by a disintegrin and metalloprotease with thrombospondin type I repeats-13 (ADAMTS13) deficiency (activity levels ≤ 10%) leading to formation of platelet thrombi, resulting in thrombocytopenia,^2^^,^^3^ to hemolytic anemia with fragmented red blood cells (schistocytes), microvessel occlusion, and tissue injury.^4^ It is suspected on clinical presentation (typically neurologic involvement with mild acute renal failure) and laboratory results (TMA syndrome). Given the high rate of early mortality, emergency TTP treatment is mandatory,^5^^,^^6^ which is based on an association of plasma therapy, corticosteroids, and targeted therapies, including rituximab^7^ and caplacizumab.^8^^,^^9^ Therapeutic plasma exchanges need to be started as soon as diagnosis is suspected.^6^

Differential diagnosis between TTP and other TMAs, and especially HUS, remains a challenge to some cases because of an overlap in clinical signs between these conditions. In TMA diagnosis workup, determination of ADAMTS13 activity is the cornerstone; however, the assay remains unavailable in many hospitals. To assist clinician decision-making, several scores have been developed to predict severe ADAMTS13 deficiency, for example, the PLASMIC score^10^ and the French score.^11^ Importantly, although revealing good predictive values, these scores have been developed in TMA cohorts characterized by a high TTP prevalence (29%–63%). These cohorts may not reflect the real prevalence of TTP among patients with TMA. Indeed, TTP prevalence was found to range from 3.2% to 5.6% in the cohorts taking into account all TMA etiologies (unselected TMA cohorts).^12^^,^^13^ Thus, the predictive value of PLASMIC and French scores still needs to be studied in “real life” TMA cohorts.

In opposition to most other TMA, TTP involves the kidneys less frequently, a characteristic that was historically used to differentiate TTP from HUS.^14^ In HUS, the kidneys are the major target of TMA, leading to glomerular microthrombosis and proteinuria. On this basis, it was recently suggested that adding proteinuria level to the French score may enable TTP and HUS to be better distinguished.^15^

Thus, the aims of this study were to evaluate the diagnostic value of the PLASMIC and French scores in “real life practice” using an unselected cohort of consecutive patients with TMA and to study the value of the modified scores that include proteinuria level.

## Methods

### Selection of Patients

Adult patients (≥18 years old) admitted to the University Hospital of Angers between January 1, 2008, and December 31, 2019, with a full biological TMA syndrome were retrospectively included in the study. A full biological TMA syndrome was defined by the concomitant association of anemia (<12 g/dl in females and 13 g/dl in males), thrombocytopenia (≤150 g/l), schistocytosis (≥0.5%), and decreased haptoglobin level (≤0.4 g/l). Patients were identified from the database of the hematological laboratory. The study protocol complied with the Ethics Committee of the Angers University Hospital (no. 2019/12).

### TMA Causes

As described earlier,^13^ medical records of patients identified with having a full biological TMA syndrome were first analyzed by 5 physicians trained in nephrology, hematology, and critical care medicine to confirm or rule out a TMA diagnosis. The second step was to identify the etiology of TMA after a hierarchical analysis, according to current classifications^16^^,^^17^ and as previously described (Supplementary Figure S1).^13^ Thus, by using this methodology, we were able to identify a cohort of consecutive patients with TMA with a full biological TMA syndrome and with all etiologies considered, which we term thereafter as “unselected cohort.”

### Data Collection and Score Assessment

Demographic, clinical, and biological data at TMA diagnosis were retrospectively retrieved. PLASMIC and French scores were calculated as described^10^^,^^11^ without considering antinuclear antibodies for the latter (Table 1). Proteinuria was collected on the day of TMA diagnosis. Acute kidney injury was defined using serum creatinine levels and the Kidney Disease Improving Global Outcomes Criteria.^18^ ADAMTS13 was collected when available. As previously described, the PLASMIC score was dichotomized into high and low intermediate risk when the score was ≥6 or ≤5, respectively.^10^ The French score was dichotomized into high and low risk when the score was 2 or ≤1, respectively.^11^

**Table 1:** PLASMIC, French and modified scores

Items	Points for PLASMIC score	Points for French score
Creatinine		
<2.0 mg/dl or <177 μmol/l	1	
<2.273 mg/dl or <200 μmol/l		1
Platelet count < 30 g/l	1	1
Hemolysis variable^a^	1	
No active cancer	1	
No history of solid-organ or stem-cell transplant	1	
MCV < 90 per 1 μm^3^	1	
INR < 1.5	1	
Modified scores		
Proteinuria level < 1.2 g/g of creatininuria	+1	+1

INR, international normalized ratio; MCV, mean corpuscular volume.

a Reticulocyte count > 2.5%, or haptoglobin undetectable, or indirect bilirubin > 2.0 mg/dl.

### Modified PLASMIC and French Score Assessment

Modified PLASMIC and French scores (Table 1) were formulated by adding 1 point when proteinuria level was <1.2 g/g of creatininuria, as determined by receiver operating characteristic curve analysis as the best threshold for TTP diagnosis (Supplementary Figure S2B). Thus, modified PLASMIC and French scores ranged from 0 to 8 and from 0 to 3, respectively. The modified PLASMIC score was dichotomized into high and low intermediate risk when the score was ≥7 or ≤6, respectively. The modified French score was dichotomized into high and low risk when the score was equal to 3 or ≤2, respectively.

### Statistical Analysis

Quantitative variables, presented as median (interquartile range), were compared with the Mann-Whitney *U* test (or Kruskal-Wallis test followed by Dunn *post hoc* test for multiple comparisons when applicable). Qualitative variables, presented as the absolute value and percentage, were compared using the χ^2^ test (or Fisher exact test when necessary). TTP diagnosis performance was analyzed using receiver operating characteristic curves. AUCs were compared using a Delong test.^19^ Performances of high-risk scores for TTP diagnosis were compared using the McNemar test (for sensitivities and specificities),^20^ generalized score statistics (for negative and positive PVs),^21^ or a regression model approach (for negative and positive likelihood ratios).^22^ Statistical analysis was performed using Prism GraphPad Software version 6.01 (Prism, La Jolla, CA) and R version 4.0. *P* < 0.05 was considered significant.

## Results

### Flow Chart of the Study

**Figure 1:**
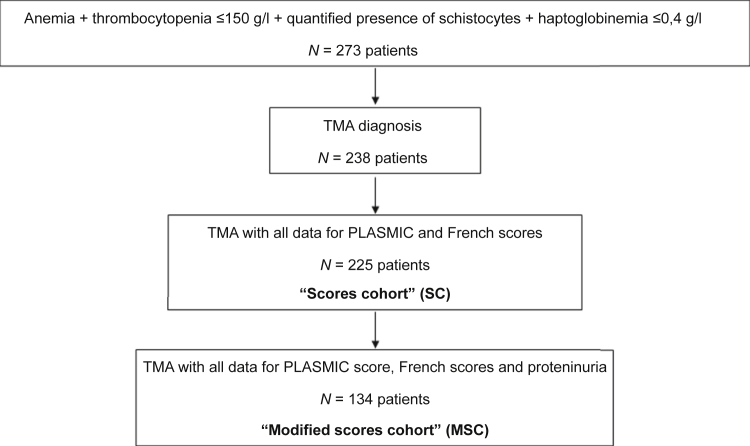
Flowchart of the study. MSC, modified score cohort; SC, score cohort; TMA, thrombotic microangiopathy.

During the above-mentioned period, we identified 485 patients with thrombocytopenia and schistocytosis (≥0.5%) and 5031 patients with haptoglobin (≤0.4 g/l). After crossing data sets, we identified 273 patients with a full biological TMA syndrome. After a medical chart review, 238 patients were finally diagnosed with having TMA (28 had no evidence of TMA), in whom 225 patients had all components of the PLASMIC and French scores (thereafter called the “scores cohort”). Proteinuria determination at diagnosis was available in 158 patients. Finally, 134 patients had all components of the PLASMIC score, the French score, and proteinuria determination at diagnosis (thereafter called the “modified scores cohort” [MSC]) (Figure 1).

### Baseline Characteristics of the Cohort

The “scores cohort” (in which TTP prevalence was 6.2%, *n* = 14) is described in Supplementary Table S1. The “modified scores cohort” (in which TTP prevalence was 7.5%, *n* = 10) is described in Table 2. ADAMTS13 assessment was available for 40 patients (29.8%) of the MSC, including all patients with TTP (ADAMTS13 < 10%). Patients with TTP had lower levels of proteinuria (0.87 g/g creatininuria [0.48–2.25] vs. 2.47 [1.06–5.07], *P* = 0.03) and a lower platelet count (16.5 g/l [9–37.25] vs. 48 [28–77], *P* < 0.001) than all other patients with TMA (Table 2).

**Table 2:** Clinical and biological presentation of TMA with data available for MSCs (*N* = 134)

MSC *N* = 134	Primary TMA *n* = 17		Secondary TMA *n* = 117								
	TTP *n* = 10	a-HUS *n* = 7	t-HUS *n* = 4	Pregnancy *n* = 67	Drugs *n* = 8	Infections *n* = 9	Active malignancy *n* = 7	Malignant HT *n* = 7	Transplantation *n* = 9	Autoimmune disease *n* = 4	Other TMA *n* = 2
Clinical characteristics											
Age, yr	52 [32–68]	41 [33–76]	65 [56–73.7]	31 [26–34]	59.5 [47.75–62.5]	63 [50.5–66]	67 [55–81]	34 [30–42]	32 [26–64]	70 [50.75–81]	80 [75–85]
Females, *n* [%]	6 [60]	7 [100]	3 [75]	67 [100]	5 [62]	3 [33]	5 [71]	2 [29]	3 [33]	2 [50]	0 [0]
Neurologic signs	7 [70]	6 [86]	3 [75]	20 [30]	1 [12]	2 [22]	3 [43]	4 [57]	2 [22]	1 [25]	0 [0]
Diarrhea	1 [10]	3 [43]	4 [100]	0 [0]	1 [12]	1 [11]	0 [0]	0 [0]	1 [11]	0 [0]	0 [0]
AKI	7 [70]	6 [86]	4 [100]	20 [30]	7 [87]	7 [78]	4 [57]	7 [100]	5 [55]	4 [100]	2 [100]
Biological presentation											
Hemoglobin, g/dl	6.35 [5.65–7.95]	8.4 [4.6–10.9]	8.3 |6.12–9.57]	9.9 [8.8–10.8]	6.85 [5.82–8.4]	7.4 [5.05–8.95]	6.2 [5.8–7.7]	6.8 [6–7.8]	7.9 [7.3–9.35]	8.55 [6.77–10.33]	7.35 [6.1–8.6]
Platelet count, g/l	16.5 [8–37.25]	74 [31–95]	39.5 [26.5–93.75]	54 [31–78]	58 [39.5–115]	17 [11–40.5]	35 [4–61]	92 [75–101]	25 [18–79.5]	111.5 [99.75–121.8]	82.5 [31–134]
LDH, IU/l	1258 [737–1787]	1535 [412–3577]	949 [475–3123]	957 [502–2041]	621.5 [413–890]	1253 [600–2909]	1958 [1319–2553]	686 [631–1630]	475 [376–666]	566 [403–576]	517.5 [240–795]
Schistocytes, *n* (%)											
0.5%–1%	1 [10]	1 [14]	0 [0]	27 [40]	3 [37]	2 [22.2]	0 [0]	3 [43]	4 [44]	0 [0]	0 [0]
1%–3%	1 [10]	3 [43]	2 [50]	36 [54]	1 [12]	3 [33.3]	0 [0]	1 [14]	4 [44]	4 [100]	1 [50]
3%–5%	1 [10]	0 [0]	1 [25]	3 [43]	3 [37]	1 [11]	4 [57]	1 [14]	1 [11]	0 [0]	1 [50]
5%–10%	4 [40]	3 [43]	1 [25]	0 [0]	1 [12]	3 [33.3]	2 [29]	1 [14]	0 [0]	0 [0]	0 [0]
>10%	3 [30]	0 [0]	0 [0]	1 [1.5]	0 [0]	0 [0]	1 [14]	1 [14]	0 [0]	0 [0]	0 [0]
Elevated free bilirubin,^a^*n* [%]	7 [70]	2 [29]	2 [50]	17 [25]	1 [12]	7 [78]	4 [57]	1 [14]	4 [44]	0 [0]	1 [50]
Elevated liver enzyme,^b^*n* [%]	4 [40]	2 [29]	3 [75]	60 [89]	0 [0]	6 [67]	6 [86]	1 [14]	4 [44]	3 [75]	0 [0]
Fibrinogen, g/l	3.61 [1.61–4.6]	5.45 [3.16–6.5]	3.64 [3.08–4.71]	4.58 [3.34–5.63]	3.39 [2.92–4.41]	3.56 [2.1–4.9]	3.56 [2.05–4.53]	4.32 [3.86–5.52]	3.7 [2.73–4.1]	3.76 [2.55–6.14]	3.25 [2.64–3.86]
Prothrombin time, %	77 [63–91]	92 [75–113]	84 [67.75–95.75]	105 [91–116]	85.5 [76.5–102.3]	63 [42.56–93.5]	85 [65–91]	96 [79–115]	91 [75–110]	61.5 [59.5–88.25]	90.5 [77–104]
C-reactive protein, mg/l	6 [3–25]	10 [4–53.75]	29 [8–113]	26 [8–55]	29 [4–98]	21 [4.5–93.75]	62 [38–168]	8 [3.5–42.5]	39 [3–50]	76 [3–169]	—
Serum creatinine, mg/dl	1.045 [0.861–2.091]	5.048 [4.33–14.114]	2.455 [1.295–6.716]	0.727 [0.614–0.955]	2.534 [1.477–5.261]	1.568 [1.063–3.091]	2.602 [0.682–6.136]	11.08 [7.307–16.693]	2.864 [0.795–5.852]	2.17 [1.705–4.875]	1.761 [1.693–1.841]
Proteinuria, g/g	0.87 [0.48–2.24]	4.8 [3.28–14.3]	2.78 [1.17–6.48]	2.52 [0.96–5.22]	5.03 [1.56–7.44]	1.5 [0.52–5.25]	1.44 [0.31–4.56]	2.9 [2.3–6.8]	2.4 [2.22–3.13]	1.38 [0.42–3.29]	0.09 [0.06–0.09]
Albuminemia, g/l	36 [29.5–39.25]	30 [26–35]	27.5 [21.5–33.5]	23 [20.25–26]	27.65 [24–31.75]	28.3 [20.5–33]	25 [23–29]	36.5 [27–40]	39 [3–50]	37 [25–40]	34.75 [27–42.5]

a-HUS, atypical hemolytic uremic syndrome; AKI, acute kidney injury; HT, hypertension; LDH, lactate dehydrogenase; MSC, modified score cohort; t-HUS, typical hemolytic uremic syndrome; TMA, thrombotic microangiopathy; TTP, thrombotic thrombocytopenic purpura.

Transplantation-associated TMA refers to stem cells and solid-organ transplantation. AKI was defined using the Kidney Disease Improving Global Outcomes Criteria; only serum creatinine criteria were used for the diagnosis.

a Elevated free bilirubin corresponds to free bilirubin >1 mg/dl.

b Elevated liver enzyme corresponds to liver enzyme ≥1 times the upper limit of normal.

### PLASMIC and French Standard Scores (in Scores Cohort)

In the “score cohort,” the PLASMIC score was higher in patients with TTP (6 [5.5–6.25]) than in all other patients with TMA (5 [4–6], *P* = 0.02) (Supplementary Table S2).

**Figure 2:**
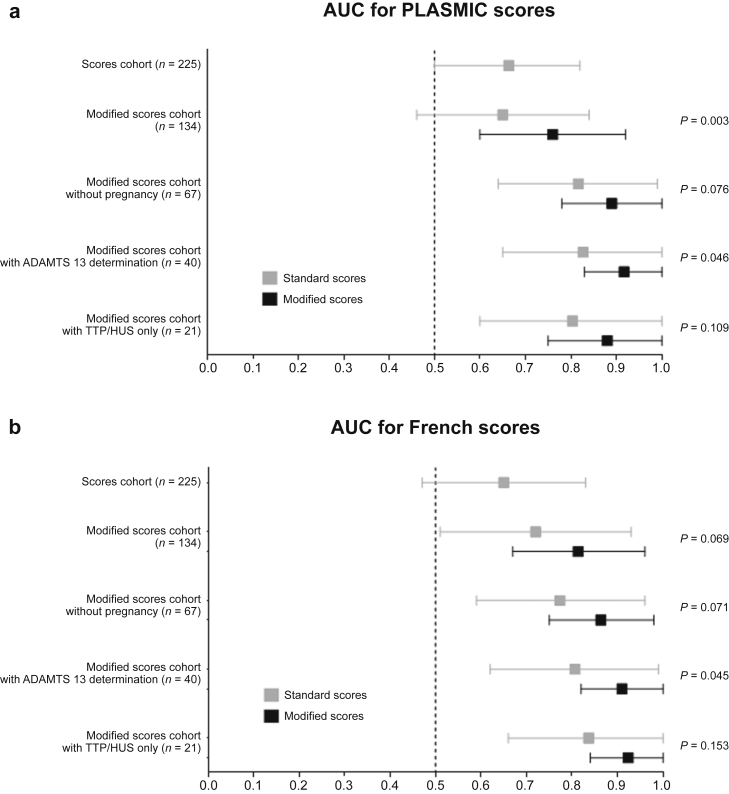
Performance of (a) PLASMIC and (b) French scores to predict TTP. *P* value refers to the comparison between the standard and modified scores. ADAMTS13, a disintegrin and metalloprotease with thrombospondin type I repeats-13; AUC, area under the receiver operating characteristic curve; HUS, hemolytic uremic syndrome; TTP, thrombotic thrombocytopenic purpura.

The ability of the PLASMIC score to distinguish TTP from other TMA diagnoses in this population was low (AUC = 0.67 [0.50–0.82], *P* = 0.02) (Figure 2 and Supplementary Figure S2A). A high-risk (score ≥ 6) predicted TTP with a sensitivity of 79%, specificity of 60%, PPV of 12%, and negative predictive value (NPV) of 98%.

In the “score cohort,” the French score tended to be higher in patients with TTP (2 [0.75–2]) than in all other patients with TMA (1 [1–1], *P* = 0.058) (Supplementary Table S2).

The ability of the French score to distinguish between TTP and other TMA diagnoses in this population was in similar ranges (AUC = 0.65 [0.47–0.83], *P* = 0.06) (Figure 2 and Supplementary Figure S2A). A high-risk (score ≥ 2) predicted TTP with a sensitivity of 57%, specificity of 81%, PPV of 16%, and NPV of 97%.

### Description of Standard and Modified PLASMIC Scores (in MSC)

In MSC, the standard PLASMIC score was similar in patients with TTP (6 [5.5–6.25]) than in all other patients with TMA (5 [5–6], *P* = 0.08). It was higher in patients with TTP than in patients with a-HUS or drug- and transplantation-associated TMA (Table 3).

**Table 3:** Standard and modified PLASMIC and French scores according to etiology of TMA (MSC, *N* = 134)

MSC *N* = 134	TTP *n* = 10	TMA (TTP excluded) *n* = 124	*P* value	a-HUS *n* = 7	t-HUS *n* = 4	Pregnancy *n* = 67	Drugs *n* = 8	Infections *n* = 9	Active malignancy *n* = 7	Malignant HT *n* = 7	Transplantation *n* = 9	Autoimmune disease *n* = 4	Other TMA *n* = 2
Proteinuria level < 1.2 g/g	8 [80]	34 [27]	0.002	1 [14]	1 [25]	20 [30]	1 [12.5]	4 [44]	4 [57]	0 [0]	2 [22]	2 [50]	2 [100]
PLASMIC score	6 [5.5–6.25]	5 [5–6]	0.08	4 [4–5]^a^	5 [4–6]	6 [6–6]	4 [4–4.75]^a^	5 [3.5–5.5]	4 [4–5]	5 [5–5]	4 [4–5]^a^	4.5 [4–5.75]	5 [5–5]
Components of the PLASMIC score													
Platelets <30 g/l, n [%]	7 [70]	31 [25]	0.006	1 [14]	1 [25]	16 [24]	0 [0]	5 [55]	3 [43]	0 [0]	5 [55]	0 [0]	0 [0]
Hemolysis,^b^*n* [%]	10 [100]	122 [98]	1	7 [100]	4 [100]	65 [97]	8 [100]	9 [100]	7 [100]	7 [100]	9 [100]	4 [100]	2 [100]
No active neoplasia, n [%]	10 [100]	112 [90]	0.6	7 [100]	4 [100]	67 [100]	7 [87]	7 [78]	0 [0]	7 [100]	7 [78]	4 [100]	2 [100]
No history of transplant, *n* [%]	9 [90]	108 [87]	1	5 [72]	3 [75]	67 [100]	7 [87]	6 [67]	7 [100]	7 [100]	0 [0]	4 [100]	2 [100]
MCV <90 per 1 μm^3^*n* [%]	5 [50]	86 [69]	0.29	4 [57]	2 [50]	55 [82]	0 [0]	3 [33]	6 [86]	6 [86]	8 [89]	2 [50]	0 [0]
INR <1.5 *n* [%]	9 [90]	116 [93]	0.51	7 [100]	4 [100]	64 [95]	8 [100]	6 [67]	6 [86]	7 [100]	8 [89]	4 [100]	2 [100]
Serum creatinine <2 mg/dl, *n* [%]	8 [80]	82 [66]	0.50	0 [0]	2 [50]	62 [93]	3 [37]	5 [55]	3 [43]	0 [0]	4 [44]	1 [25]	2 [100]
PLASMIC score risk			0.04										
Low intermediate (≤5), *n* [%]	2 [20]	66 [53]		7 [100]	2 [50]	16 [24]	8 [100]	7 [78]	6 [86]	7 [100]	8 [89]	3 [75]	2 [100]
High (≥6), *n* [%]	8 [80]	58 [47]		0 [0]	2 [50]	51 [76]	0 [0]	2 [22]	1 [14]	0 [0]	1 [11]	1 [25]	0 [0]
Modified PLASMIC score	7 [5.75–7]	6 [5–6]	<0.001	5 [4–5]^a^	5 [4–6.75]	6 [6–7]	4 [4–4.75]^a^	5 [4–6]^a^	5 [4–6]^a^	5 [5–5]^a^	4 [4–5]^a^	5 [4.25–6.5]	6 [6–6]
Modified PLASMIC score risk			<0.001										
Low intermediate (≤6), *n* [%]	3 [30]	102 [82]		7 [100]	3 [75]	47 [70]	8 [100]	9 [100]	7 [100]	7 [100]	9 [100]	3 [75]	2 [100]
High (≥7), *n* [%]	7 [70]	22 [18]		0 |0]	1 [25]	20 [30]	0 [0]	0 [0]	0 [0]	0 [0]	0 [0]	1 [25]	0 [0]
French score	2 [0.75–2]	1 [1–1]	0.01	0 [0–0]^a^	1 [0.25–1]	1 [1–1]	0 [0–1]^a^	1 [0.5–2]	1 [0–1]	0 [0–0]^a^	1 [0–2]	0.5 [0–1]	1 [1–1]
Components of the French score													
Platelets <30 g/l, *n* [%]	7 [70]	31 [25]	0.006	1 [14]	1 [25]	16 [24]	0 [0]	5 [55]	3 [43]	0 [0]	5 [55]	0 [0]	0 [0]
Serum creatinine <2.273 mg/dl, *n* [%]	8 [80]	83 [67]	0.5	0 [0]	2 [50]	62 [92]	3 [37]	5 [55]	3 [43]	0 [0]	4 [44]	2 [50]	2 [100]
French score risk			< 0.001										
Low (≤1), *n* [%]	3 [30]	104 [84]		7 [100]	4 [100]	54 [81]	8 [100]	6 [67]	6 [86]	7 [100]	6 [67]	4 [100]	2 [100]
High (= 2), *n* [%]	7 [70]	20 [16]		0 [0]	0 [0]	13 [19]	0 [0]	3 [33]	1 [14]	0 [0]	3 [33]	0 [0]	0 [0]
Modified French score	2.5 [1.75–3]	1 [1–2]	<0.001	0 [0–1]^a^	1 [0.25–1.75]	1 [1–2]	0 [0–1]^a^	2 [1–2]	2 [0–2]	0 [0–0]^a^	1 [0–2]^a^	1 [0.25–1.75]	2 [2–2]
Modified French score risk			< 0.001										
Low (≤2), *n* [%]	5 [50]	119 [96]		7 [100]	4 [100]	63 [94]	8 [100]	8 [89]	7 [100]	7 [100]	9 [100]	4 [100]	2 [100]
High (= 3), *n* [%]	5 [50]	5 [4]		0 [0]	0 [0]	4 [6]	0 [0]	1 [11]	0 [0]	0 [0]	0 [0]	0 [0]	0 [0]

a-HUS, atypical hemolytic uremic syndrome; INR, international normalized ratio; HT, hypertension; MCV, mean corpuscular volume; MSC, modified score cohort; t-HUS, typical hemolytic uremic syndrome; TMA, thrombotic microangiopathy; TTP, thrombotic thrombocytopenic purpura.

Transplantation-associated TMA refers to stem cells and solid-organ transplantation.

The *P* value column refers to the test between TTP and TMA without TTP.

a Refers to Dunn’s post-test <0.05 for pairwise comparisons of patients with TTP and those in other diagnostic categories (*P* value for previous Kruskal-Wallis was <0.0001). These tests were performed only for standard and modified scores (not components nor risk categories).

b Reticulocyte count >2.5%, or haptoglobin undetectable, or indirect bilirubin >2.0 mg/dl.

To build the modified PLASMIC score, we chose to add 1 point (in case of proteinuria level < 1.2 g/g) to the standard scores because it performed better than adding more (Supplementary Figure S3A–C).

The modified PLASMIC score was higher in patients with TTP (7 [5.75–7]) than in all other patients with TMA (6 [5–6], *P* = 0.004). It was also higher than in patients with a-HUS or drug-, infection-, active malignancy-, transplantation-, and malignant hypertension-associated TMA (Table 3).

### Performances of Standard and Modified PLASMIC Scores (in MSC)

**Figure 3:**
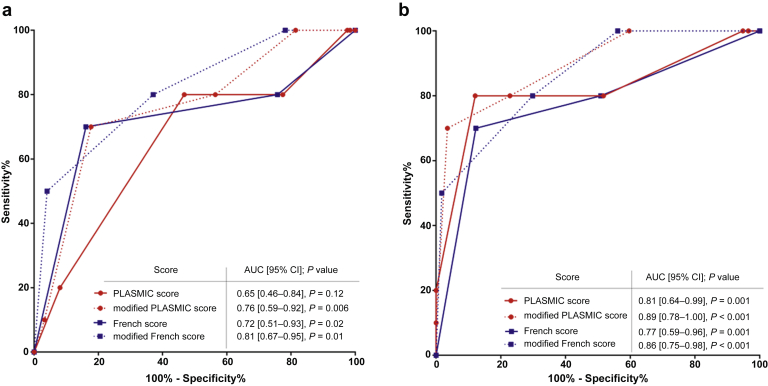
Performance of PLASMIC and French scores to predict TTP in (a) MSC and (b) MSC without pregnancy. MSC included 134 patients (with 10 patients with TTP), and MSC without pregnancy included 67 patients (with 10 patients with TTP). *P* value refers to the comparison between AUC and 0.5. AUC, area under the receiver operating characteristic curve; MSC, modified score cohort; TTP, thrombotic thrombocytopenic purpura.

In MSC, the AUC of the PLASMIC score was 0.65 ([0.46–0.84], *P* = 0.12). The AUC of the modified PLASMIC score was 0.76 ([0.59–0.92, *P* = 0.006]), which was significantly higher than the standard score (*P* = 0.003) (Figures 2a and 3a).

When dichotomized into high (score ≥7) and low intermediate risk, the modified PLASMIC (vs. standard) score predicted TTP with a similar sensitivity (70% [42–98] vs. 80% [55–100], *P* = 0.32), a better specificity (82% [76–89] vs. 53% [44–62], *P* < 0.001), a better PPV (24% [9–40] vs. 12% [4–20], *P* = 0.015), and a similar NPV (97% [94–100] vs. 97% [93–100], *P* = 0.94) (Table 4, upper panel). Of note, using a nonmodified threshold for high-risk patients (score ≥ 6) resulted in lower performance (Supplementary Table S3).

**Table 4:** Performance of high-risk standard and modified scores to predict TTP

MSC *N* = 134	Standard score	Modified score	*P* value
PLASMIC score	≥6	≥7	
Sensitivity	80% [55–100]	70% [42–98]	0.32
Specificity	53% [44–62]	82% [76–89]	<0.001
PPV	12% [4–20]	24% [9–40]	0.015
NPV	97% [93–100]	97% [94–100]	0.94
LR+	1.7 [1.2–2.5]	3.9 [2.3–6.9]	<0.001
LR−	0.4 [0.1–1.3]	0.4 [0.1–0.9]	0.94
French score	= 2	= 3	
Sensitivity	70% [42–98]	50% [19–81]	0.16
Specificity	84% [77–90]	96% [93–99]	<0.001
PPV	26% [9–42]	50% [19–81]	0.046
NPV	97% [94–100]	96% [93–99]	0.28
LR+	4.3 [2.5–7.7]	12.4 [4.3–35.8]	0.02
LR−	0.4 [0.1–0.9]	0.5 [0.3–1.0]	0.30

MSC (without pregnancy) *N* = 67	Standard score	Modified score	*P* value
PLASMIC score	≥ 6	≥ 7	
Sensitivity	80% [55–100]	70% [42–98]	0.32
Specificity	88% [79–96]	96% [92–100]	0.025
PPV	53% [28–79]	78% [51–100]	0.031
NPV	96% [91–100]	95% [89–100]	0.43
LR+	6.5 [3.0–13.9]	20.0 [4.8–82.6]	0.067
LR−	0.2 [0.1–0.8]	0.3 [0.1–0.8]	0.45
French score	= 2	= 3	
Sensitivity	70% [42–98]	50% [19–81]	0.16
Specificity	88% [79–96]	98% [95–100]	0.014
PPV	50% [24–76]	83% [54–100]	0.029
NPV	94% [88–100]	92% [85–99]	0.25
LR+	5.7 [2.6–12.7]	28.5 [3.7–219.0]	0.09
LR−	0.3 [0.1–0.9]	0.5 [0.3–0.9]	0.28

LR+, positive likelihood ratio; LR−, negative likelihood ratio; MSC, modified score cohort; NPV, negative predictive value; PPV, positive predictive value; TTP, thrombotic thrombocytopenic purpura.

In MSC, *N* = 134 (upper panel) or after pregnancy exclusion, *n* = 67 (lower panel).

When compared with patients with low intermediate-risk score, patients with a high-risk modified PLASMIC score were more likely to have TTP than patients with a high-risk standard score (positive likelihood ratio 3.9 [2.3–6.9] vs. 1.7 [1.2–2.5], *P* < 0.001) (Table 4, upper panel).

In other words, among the 10 patients with TTP diagnosis in MSC, 8 patients (80%) versus 7 patients (70%) were classified in the high-risk group according to the standard or modified PLASMIC score, respectively (*P* = 1). More importantly, the modified PLASMIC score identified significantly more patients with a TMA of any other etiology (patients with non-TTP) (vs. the standard score): 102 (82%) versus 66 (53%) were in the low intermediate-risk group (*P* < 0.001) (Table 3).

### Description of Standard and Modified French Scores (in MSC)

The standard French score was higher in patients with TTP (2 [0.75–2]) than in all other patients with TMA (1 [1–1], *P* = 0.01). It was also higher in patients with TTP than in patients with a-HUS or drug- and malignant hypertension-associated TMA (Table 3).

To build the modified French score, we chose to add 1 point (in case of proteinuria level < 1.2 g/g) to the standard scores because, as for the PLASMIC score, it performed better than adding more (Supplementary Figure S3D–F).

The modified French score was higher in patients with TTP (2.5 [1.75–3]) than in all other patients with TMA (1 [1–2], *P* = 0.0002). It was also higher than in patients with a-HUS or drug-, transplantation-, and malignant hypertension-associated TMA (Table 3).

### Performances of Standard and Modified French Scores (in MSC)

The modified French score had a higher AUC (0.81 ([0.67–0.96], *P* = 0.001) than the standard French score (0.72 ([0.51–0.93], *P* = 0.02), although this did not reach statistical significance (*P* = 0.069) (Figures 2b and 3a).

When dichotomized into high (score = 3) and low risks, the modified French score (vs. the standard score) predicted TTP with a similar sensitivity (50% [19–81] vs. 70% [42–98], *P* = 0.16), a better specificity (96% [93–99] vs. 84% [77–90], *P* < 0.001), a better PPV (50% [19–81] vs. 26% [9–42], *P* = 0.046), and a similar NPV (96% [93–99] vs. 97% [94–100], *P* = 0.28) (Table 4, upper panel). Of note, using a nonmodified threshold for high-risk patients (score ≥ 2) resulted in lower performance (Supplementary Table S3).

When compared with patients with low-risk score, patients with a high-risk modified French score were more likely to have TTP than patients with a high-risk standard score (positive likelihood ratio 12.4 [4.3–35.8] vs. 4.3 [2.5–7.7], *P* = 0.02) (Table 4, upper panel).

In other words, 7 patients (70%) versus 5 patients (50%) with TTP were classified in the high-risk group according to the standard or modified French score, respectively (*P* = 0.65). In parallel, 104 patients (84%) versus 119 patients (96%) with non-TTP with a TMA of any other etiology were in the low intermediate-risk group according to the standard or modified French score (*P* = 0.002) (Table 3).

### Both Standard and Modified Scores Performed Better in Selected Subpopulations

After the exclusion of pregnant women, for whom the cause of TMA is often clear, all these scores, modified or not, were improved (Figure 3b). The addition of proteinuria level tended to improve the standard scores (*P* = 0.076 and 0.071 for the AUC of the modified vs. standard PLASMIC and French scores, respectively; Figure 2). Again, it improved the specificity and PPV of high-risk patients for TTP diagnosis, with a trend for a better positive likelihood ratio (Table 4, lower panel).

It is worth noting that these modified scores were also better when considering the subpopulation with ADAMTS13 determination (*n* = 40; Figure 2 and Supplementary Figure S4A) or only TTP and all patients with HUS (*n* = 21; Figure 2 and Supplementary Figure S4B).

## Discussion

Clinicians need to rapidly identify patients with primary TMA and especially those with TTP, for whom therapeutic plasma exchanges or fresh frozen plasma administration must be initiated as soon as possible. Scores have therefore been developed to help identify these patients with TTP. It must be noted that these scores have been evaluated and validated in cohorts with a high TTP prevalence. Nevertheless, as recently reported, these populations do not accurately reflect clinical practice.^12^^,^^13^ Here, we reveal that PLASMIC and French scores, when applied to an unselected TMA population characterized by a low TTP prevalence, do not effectively identify TTP from other TMA etiologies. Moreover, our results suggest that incorporating proteinuria level into the established scores may improve their predictive value.

Bendapudi *et al.*^10^ created the PLASMIC score using 2 cohorts with a high prevalence of TTP (14%–47%) and revealed its accuracy for TTP diagnosis (AUC = 0.91–0.96). Further studies with a high TTP prevalence (25%–70%) confirmed the good diagnostic value of both the PLASMIC and French scores.^23^, ^24^, ^25^, ^26^, ^27^, ^28^, ^29^ Nevertheless, they also included highly selected patients recruited among those who had ADAMTS13 measurement.^30^ As a consequence, the TTP prevalence was much higher in these study groups than TTP prevalence in clinical practice. Interestingly, a recent meta-analysis evaluated the validity of the PLASMIC score^30^ and revealed that a score < 5 was associated with high sensitivity and NPV, suggesting it could be used to rule out TTP and to exclude the need for emergency therapeutic plasma exchanges. This was also the case in our cohort, but at the price of a strong drop in specificity (Supplementary Table S4). In this meta-analysis, which included studies with a median TTP prevalence of 35%, the PPV of the PLASMIC score decreased when TTP prevalence was lower (for a 10% TTP prevalence and PLASMIC score ≥ 5, the PPV decreased to 21% and the NPV was 100%). In our study, applied to an unselected TMA population with a low TTP prevalence, the standard PLASMIC score was not very successful in predicting TTP.

The diagnostic score proposed by Coppo *et al.*,^11^ namely the French score, has the advantage of being more simple to calculate than the PLASMIC score. Using 3 criteria (platelets < 30 g/l, serum creatinine level < 2.273 mg/dl, and, to a lesser extent, positivity for antinuclear antibodies), it was found that the score predicts ADAMTS13 deficiency with a sensitivity of 99%, specificity of 48%, PPV of 85%, and NPV of 93%.^11^ Applied to the PLASMIC cohort, the French score had an AUC of 0.88 ([0.83–0.91], *P* = 0.003) and the high-risk group (presence of 2 criteria) identified 83% of patients with severe ADAMTS13 deficiency.^10^ Nevertheless, as observed for the PLASMIC score, when applied to our cohort, the French score was also not very successful in predicting a TTP diagnosis. This observation was predictable as the French score was developed in a TMA cohort that voluntarily excluded patients with secondary TMA.^11^ Interestingly, a recent study revealed a decreased sensitivity and specificity of PLASMIC and French scores in older patients (>60 years old)^31^ as compared with younger patients. The lower diagnostic value of these scores may be related to less typical presentations of TTP in older patients, as suggested by the authors, but it may also be related to the lower TTP prevalence and enrichment with secondary TMA causes in old patients.

In this study, we reveal that integrating proteinuria level, a marker of renal injury unlikely to be observed in TTP, improved the performance of both the PLASMIC and French scores. When set to ≥7, the modified high-risk PLASMIC score revealed a significant increase in specificity, PPV, and positive likelihood ratio for TTP diagnosis, at the cost of a slight, but not significant, decrease in sensitivity. In the same way, proteinuria level also improved the performance of a high-risk French score (score = 3) with an increase in specificity, PPV, and positive likelihood ratio for TTP diagnosis when compared with the standard score. In practice, this means that it will be easier to identify patients with non-TTP. Thus, by reducing the false positive rate, the modified scores could be used as part of cost-saving strategies, by reducing unnecessary therapeutic plasma exchanges and inappropriate use of rituximab and/or caplacizumab.^32^ Despite not being statistically significant, the loss of sensitivity (and the subsequent false-negative risk) of the modified scores in relation to the standard scores should be taken into consideration and reminds us that clinical expertise cannot be replaced simply by applying a statistical score.^33^

Our study has several limitations, the first of which being its retrospective design. Nevertheless, the low incidence of TTP makes it difficult to carry out prospective studies. Second, we enrolled all patients with data available to calculate the PLASMIC and French scores, but not all patients had an ADAMTS13 assay available, which is the gold standard for TTP diagnosis. Nevertheless, all our patients with TTP had undetectable ADAMTS13 levels and it is known that the mortality of untreated patients with TTP is close to 90%.^34^ Thus, it is reasonable to exclude TTP diagnosis in patients for whom no ADAMTS13 assay was performed as we have follow-up data regarding these patients. In addition, the performance of the modified scores with proteinuria level was significantly better in the subpopulation where ADAMTS13 levels were available. Third, we used a French score without antinuclear antibodies because this test was rarely performed in our cohort. Nevertheless, previous studies have revealed that a French score based on both platelet count and serum creatinine has a high predictive value for TTP in selected populations.^10^^,^^31^

In conclusion, we have revealed that PLASMIC and French scores are not effective tools for assisting in TTP diagnosis when applied to an unselected TMA population with a low TTP prevalence. The inclusion of proteinuria level in the scores may improve their performance, especially specificity, PPV, and positive likelihood ratio.

To confirm our results, testing these modified scores in larger, multicenter cohorts, ideally prospective, of patients with TMA for all of which ADAMTS13 is known, would be of great interest. Then, if all the studies converge to the same results, it would be interesting to consider a prospective study where the initial TMA management would be guided by the results of the modified scores. The modified scores could indeed help clinicians in deciding on the emergency use of TPE and could lead to cost savings. Thus, we believe that these modified scores could have an important place in the algorithm for the management of patients with suspected TTP recently proposed by Coppo *et al.*^35^

## Disclosure

All the authors declared no competing interests.

## Supplementary Material

Supplementary File (PDF)

**Figure S1.** Hierarchical analysis to identify etiology of thrombotic microangiopathy.

**Figure S2.** Performance of PLASMIC and French scores to predict TTP in SC (A). Determination of the best proteinuria threshold for predicting a TTP diagnosis in MSC (B).

**Figure S3.** Performance of PLASMIC (A–C) and French (D–F) scores in predicting a diagnosis of TTP considering various modified scores.

**Figure S4.** Performance of PLASMIC and French scores in predicting a diagnosis of TTP, considering patients with TTP and HUS (A) and patients with an ADAMTS13 determination available (B).

**Table S1.** Clinical and biological presentation of TMA with PLASMIC and French scores available (in SC, n = 225).

**Table S2.** PLASMIC score, French score, and components according to the different etiologies of TMA (in SC, n = 225).

**Table S3.** Performance of high-risk standard and modified scores to predict TTP according to adjusted or unadjusted threshold.

**Table S4.** Performance of high-risk standard PLASMIC scores with standard (≥6) or modified (≥5) threshold to predict TTP (in SC, n = 225).

## References

[1] Allford S.L., Hunt B.J., Rose P., Machin S.J., Haemostasis and Thrombosis Task Force, British Committee for Standards in Haematology (2003). Guidelines on the diagnosis and management of the thrombotic microangiopathic haemolytic anaemias. Br J Haematol.

[2] Moake J.L., Rudy C.K., Troll J.H. (1982). Unusually large plasma factor VIII:von Willebrand factor multimers in chronic relapsing thrombotic thrombocytopenic purpura. N Engl J Med.

[3] Levy G.G., Nichols W.C., Lian E.C. (2001). Mutations in a member of the ADAMTS gene family cause thrombotic thrombocytopenic purpura. Nature.

[4] Kremer Hovinga J.A., Coppo P., Lämmle B. (2017). Thrombotic thrombocytopenic purpura. Nat Rev Dis Primers.

[5] George J.N. (2010). How I treat patients with thrombotic thrombocytopenic purpura: 2010 [published correction appears in *Blood*. 2011;117:5551]. Blood.

[6] Pereira A., Mazzara R., Monteagudo J. (1995). Thrombotic thrombocytopenic purpura/hemolytic uremic syndrome: a multivariate analysis of factors predicting the response to plasma exchange. Ann Hematol.

[7] Froissart A., Buffet M., Veyradier A. (2012). Efficacy and safety of first-line rituximab in severe, acquired thrombotic thrombocytopenic purpura with a suboptimal response to plasma exchange. Experience of the French Thrombotic Microangiopathies Reference Center. Crit Care Med.

[8] Peyvandi F., Scully M., Kremer Hovinga J.A. (2016). Caplacizumab for acquired thrombotic thrombocytopenic purpura. N Engl J Med.

[9] Scully M., Cataland S.R., Peyvandi F. (2019). Caplacizumab treatment for acquired thrombotic thrombocytopenic purpura. N Engl J Med.

[10] Bendapudi P.K., Hurwitz S., Fry A. (2017). Derivation and external validation of the PLASMIC score for rapid assessment of adults with thrombotic microangiopathies: a cohort study. Lancet Haematol.

[11] Coppo P., Schwarzinger M., Buffet M. (2010). Microangiopathies for the FRC for T: predictive features of severe acquired ADAMTS13 deficiency in idiopathic thrombotic microangiopathies: the French TMA reference center experience. PLoS One.

[12] Bayer G., Tokarski F. von, Thoreau B. (2019). Etiology and outcomes of thrombotic microangiopathies. Clin J Am Soc Nephrol.

[13] Henry N., Mellaza C., Fage N. (2021). Retrospective and systematic analysis of causes and outcomes of thrombotic microangiopathies in routine clinical practice: an 11-year study. Front Med (Lausanne).

[14] Coppo P., Bengoufa D., Veyradier A. (2004). Severe ADAMTS13 deficiency in adult idiopathic thrombotic microangiopathies defines a subset of patients characterized by various autoimmune manifestations, lower platelet count, and mild renal involvement. Medicine (Baltimore).

[15] Burguet L., Taton B., Prezelin-Reydit M. (2019). Le rapport protéinurie/créatinurie améliore nettement la discrimination SHU/PTT à l’ère des thérapies ciblées: un test simple et performant. Néphrologie Ther.

[16] Scully M., Cataland S., Coppo P. (2017). Consensus on the standardization of terminology in thrombotic thrombocytopenic purpura and related thrombotic microangiopathies. J Thromb Haemost.

[17] Fakhouri F., Zuber J., Frémeaux-Bacchi V., Loirat C. (2017). Haemolytic uraemic syndrome [published correction appears in *Lancet*. 2017;390:648]. Lancet.

[18] Kellum J.A., Lameire N., KDIGO AKI Guideline Work Group (2013). Diagnosis, evaluation, and management of acute kidney injury: a KDIGO summary (part 1). Crit Care.

[19] DeLong E.R., DeLong D.M., Clarke-Pearson D.L. (1988). Comparing the areas under two or more correlated receiver operating characteristic curves: a nonparametric approach. Biometrics.

[20] McNEMAR Q. (1947). Note on the sampling error of the difference between correlated proportions or percentages. Psychometrika.

[21] Moskowitz C.S., Pepe M.S. (2006). Comparing the predictive values of diagnostic tests: sample size and analysis for paired study designs. Clin Trials.

[22] Gu W., Pepe M.S. (2009). Estimating the capacity for improvement in risk prediction with a marker. Biostatistics.

[23] Tiscia G.L., Ostuni A., Cascavilla N. (2018). Validation of PLASMIC score and follow-up data in a cohort of patients with suspected microangiopathies from Southern Italy. J Thromb Thrombolysis.

[24] Tang N., Wang X., Li D., Sun Z. (2018). Validation of the PLASMIC score, a clinical prediction tool for thrombotic thrombocytopenic purpura diagnosis, in Chinese patients. Thromb Res.

[25] Oliveira D.S., Lima T.G., Benevides F.L.N. (2019). Plasmic score applicability for the diagnosis of thrombotic microangiopathy associated with ADAMTS13-acquired deficiency in a developing country. Hematol Transfus Cell Ther.

[26] Moosavi H., Ma Y., Miller M.J., Duncan A. (2020). Validation of PLASMIC score: an academic medical center case series (2012-present). Transfusion.

[27] Jajosky R., Floyd M., Thompson T., Shikle J. (2017). Validation of the PLASMIC score at a University Medical Center. Transfus Apher Sci.

[28] Wynick C., Britto J., Sawler D. (2020). Validation of the PLASMIC score for predicting ADAMTS13 activity <10% in patients with suspected thrombotic thrombocytopenic purpura in Alberta, Canada. Thromb Res.

[29] Li A., Khalighi P.R., Wu Q., Garcia D.A. (2018). External validation of the PLASMIC score: a clinical prediction tool for thrombotic thrombocytopenic purpura diagnosis and treatment. J Thromb Haemost.

[30] Paydary K., Banwell E., Tong J., Chen Y., Cuker A. (2020). Diagnostic accuracy of the PLASMIC score in patients with suspected thrombotic thrombocytopenic purpura: a systematic review and meta-analysis. Transfusion.

[31] Liu A., Dhaliwal N., Upreti H. (2021). Reduced sensitivity of PLASMIC and French scores for the diagnosis of thrombotic thrombocytopenic purpura in older individuals. Transfusion.

[32] Upadhyay V.A., Geisler B.P., Sun L. (2019). Utilizing a PLASMIC score-based approach in the management of suspected immune thrombotic thrombocytopenic purpura: a cost minimization analysis within the Harvard TMA Research Collaborative. Br J Haematol.

[33] Jamme M., Rondeau E. (2017). The PLASMIC score for thrombotic thrombocytopenic purpura. Lancet Haematol.

[34] Amorosi E.L., Ultmann J.E. (1966). Thrombotic thrombocytopenic purpura: report of 16 cases and review of the literature. Medicine (Baltimore).

[35] Coppo P., Cuker A., George J.N. (2019). Thrombotic thrombocytopenic purpura: toward targeted therapy and precision medicine. Res Pract Thromb Haemost.

