# Supplementary material for: Proteinuria Increases the PLASMIC and French Scores Performance to Predict Thrombotic Thrombocytopenic Purpura in Patients With Thrombotic Microangiopathy Syndrome
Source: Kidney Int Rep. 2021 Nov 16;7(2):221–31. doi: 10.1016/j.ekir.2021.11.009 (PMC8820983; doi:10.1016/j.ekir.2021.11.009)

SUPPLEMENTARY MATERIAL

Supplementary Tables

Supplementary Figures

Supplementary Tables

Supplementary Table 1: Clinical and biological presentation of TMA with PLASMIC and French scores available (in SC, n = 225).

a-HUS: atypical hemolytic uremic syndrome; AKI: acute kidney injury; LDH: Lactate dehydrogenase; Malignant HT: malignant hypertension; t-HUS: typical hemolytic uremic syndrome; TMA: thrombotic microangiopathy; TTP: thrombotic thrombocytopenic purpura.

Transplantation associated TMA refers to stem cells and solid organ transplantation.

† Elevated free bilirubin corresponds to free bilirubin > 1 mg/dL

± Elevated liver enzyme corresponds to liver enzyme ≥ 1 time the upper limit of normal.

Supplementary Table 2: PLASMIC Score, French Score and components according to the different etiology of TMA (in SC, n = 225).

a-HUS: atypical hemolytic uremic syndrome; INR: international normalized ratio; Malignant HT: malignant hypertension; MCV: mean corpuscular volume; t-HUS: typical hemolytic uremic syndrome; TMA: thrombotic microangiopathy; TTP: thrombotic thrombocytopenic purpura.

Transplantation associated TMA refers to stem cells and solid organ transplantation.

The P-value column refers to the test between TTP and TMA without TTP.

Hemolysis†: Reticulocyte count >2.5%, or haptoglobin undetectable, or indirect bilirubin >2.0 mg/dL

* refers to Dunn's post-test < 0.05 for pairwise comparisons of patients with TTP and those in other diagnostic categories (P-value for prior Kruskall-Wallis was < 0.0001). These tests were performed only for standard and modified scores (not components nor risk categories).

Supplementary Table 3: Performance of high-risk standard and modified scores to predict TTP according to adjusted or unadjusted threshold.

In MSC (Modified Score Cohort), n = 134 (upper panel) or after pregnancy exclusion, n = 67 (lower panel). NPV: negative predictive value, PPV: positive predictive value.

Supplementary Table 4: Performance of high-risk standard PLASMIC scores with standard (≥ 6) or modified (≥ 5) threshold to predict TTP (in SC, n = 225).

In SC (Score Cohort), n = 225. NPV: negative predictive value; PPV: positive predictive value.

Supplementary Figures

Supplementary Figure 1: Hierarchical analysis to identify etiology of thrombotic microangiopathy.

* Two patients were finally classified as having a-HUS in front of complement alternative pathway abnormalities.

** Other secondary causes: infections, malignant hypertension, active malignancy, drugs, transplantation or auto-immune disease related TMA.

ADAMTS13: A Disintegrin And Metalloprotease with Thrombospondin type I repeats-13; a-HUS: atypic Hemolytic Uremic Syndrome; AKI: acute kidney injury; cAP alternative pathway of complement; HELLP: Hemolysis, Elevated Liver enzyme and Low Platelets; t-HUS: typical hemolytic uremic syndrome; TMA: thrombotic microangiopathy; TTP: thrombotic thrombocytopenic purpura.

Supplementary Figure 2: Performance of PLASMIC and French scores to predict TTP in SC (A). Determination of the best proteinuria threshold for predicting a TTP diagnosis in MSC (B).

AUC: area under the curve, CI: confident interval, Se: sensibility, Sp: specificity, TTP: thrombotic thrombocytopenia purpura.

P-values refer to the comparison between AUC and 0.5.

Supplementary Figure 3: Performance of PLASMIC (A-C) and French (D-F) scores in predicting a diagnosis of TTP considering various modified scores.

ROC curves were built considering the “modified score cohort” (A, D), the “modified score cohort without pregnancy” (B, E) or only the population with an ADAMTS13 determination (C, F).

AUC: area under the curve. P-values refer to the comparison between AUC of modified scores (+1, +2 or +3 points for proteinuria <1.2 g/g, depicted in red, green, orange, respectively) and AUC of standard scores (blue).

Supplementary Figure 4: Performance of PLASMIC and French scores in predicting a diagnosis of TTP, considering TTP and HUS patients (A) and patients with an ADAMTS13 determination available (B).

(A) TTP (n = 10), HUS (n = 11). (B) ADAMTS13 available for 40 patients.

ADAMTS13: A Disintegrin And Metalloprotease with Thrombospondin type I repeats-13; AUC: area under the curve, CI: confident interval, Se: sensibility, Sp: specificity, TTP: thrombotic thrombocytopenia purpura.

P value refers to the comparison between AUC and 0.5.

Supplementary Table 1

**SC** **N = 225**	Primary TMA n= 25		Secondary TMA n= 200								
	TTP N = 14	a-HUS n = 11	t-HUS n = 8	Pregnancy n = 95	Drugs n = 12	Infections n = 20	Active malignancy n = 22	Malignant HT n =11	Transplantation n = 19	Auto-immune disease n = 6	Other TMA n = 7
Clinical characteristics											
Age, years	53.5 [43.25-65]	41 [33-76]	62.5 [49.5-73.75]	30 [27-33]	59.5 [46.5-62.5]	63.5 [49.25-72]	65.6 [59.5-75]	34 [30-48]	51 [30-63]	70 [55.5-81.75]	75 [52-89]
Females, N [%]	8 [57]	9 [82]	6 [75]	95 [100]	6 [50]	10 [50]	14 [64]	4 [36]	9 [47]	3 [50]	2 [29]
Neurological signs	11 [79]	8 [73]	7 [87]	35 [39]	2[17]	6 [30]	13 [59]	6 [54]	7 [37]	1 [17]	3 [49]
Diarrhea	2 [14]	3 [27]	8 [100]	0 [0]	2 [17]	3 [15]	22 [100]	0 [0]	5 [26]	0 [0]	1 [14]
AKI	11 [79]	10 [91]	8 [100]	28 [29]	8 [67]	15 [75]	9 [41]	9 [82]	9 [47]	6 [100]	4 [57]
Biological presentation											
Hemoglobin, g/dL	6.65 [5.67-8.75]	8.4 [6.3-9.9]	8.6 [7.1-10.13]	9.8 [8.5-10.7]	7.15 [6.32-8.85]	8.55 [7.1-9.9]	7.3 [6.1-8.05]	6.8 [6.2-7.8]	7.9 [7.5-9.5]	8.25 [6.47-9.77]	7.2 [6.1-8]
Platelet count, G/L	15 [9-37.25]	62 [31-95]	54.5 [37.25-71.25]	51 [31-77]	41 [19.75-101]	23.5 [13-40.75]	42.5 [15.75-82.25]	87 [74-97]	27 [19-41]	102.5 [71.75-117.3]	57 [32-115]
LDH, UI/L	1457 [902-1787]	867 [412-1919]	1266 [613- 1913]	895 [501-2045]	633 [473-890]	1253 [608-2988]	1075 [554-2403]	686 [473-1282]	600 [392-854]	566 [456-1447]	529 [322-1071]
Schistocytes, n (%)											
0.5-1%	1 [7]	2 [18]	0 [0]	41 [43]	5 [42]	6 [30]	2 [9]	5 [45]	8 [42]	1 [17]	3 [43]
1%-3%	3 [21]	5 [45]	3 [37]	46 [48]	3 [25]	8 [40]	7 [32]	3 [27]	7 [37]	4 [67]	2 [29]
3-5%	2 [14]	1 [9]	3 [37]	5 [5]	3 [25]	2 [10]	6 [27]	1 [9]	2 [10]	1 [17]	2 [29]
5-10%	5 [36]	3 [27]	2 [25]	2 [2]	1 [8]	3 [15]	6 [27]	1 [9]	2 [10]	0 [0]	0 [0]
> 10%	3 [21]	0 [0]	0 [0]	1 [1]	0 [0]	1 [5]	1 [4]	1 [9]	0 [0]	0 [0]	0 [0]
Elevated free bilirubin †, N [%]	10 [71]	3 [27]	2 [25]	45 [47]	0 [0]	13 [65]	0 [0]	2 [18]	6 [32]	0 [0]	4 [57]
Elevated liver enzyme ±, N [%]	4 [29]	2 [18]	6 [75]	81 [85]	3 [25]	17 [85]	16 [0]	1 [9]	9 [47]	4 [67]	2 [29]
Fibrinogen, g/L	4.27 [2.06-5.15]	4.79 [4.04-6.5]	3.86 [3.28-6.63]	4.44 [3.19-5.31]	3.74 [3.02-4.7]	3.61 [1.9-4.33]	2.36 [1.54-4.33]	4.015 [3.43-4.8]	3.16 [2.33-4.16]	3.36 [2.74-5.05]	3.2 [2.55-4.07]
Prothrombin time, %	77 [64.5-87.25]	92 [68-113]	95 [66.5-99]	104 [92-113]	88 [76-103]	59.5 [50-81.75]	76 [68.75-88.75]	96 [83-114]	88 [72-94]	61.5 [60.5-99]	77 [61-86]
C-Reactive protein, mg/L	15.5 [3.75-39.25]	10.0 {4-50]	30 [9.5-87]	23 [5.5-38.5]	33 [8.5-94]	114 [33-322]	39 [11-141]	8 [3.25-27.75]	9 [3-50]	76 [3-169]	18 [10-32.5]
Serum creatinine, mg/dL	1.443 [0.977-3.886]	4.352 [3.918-14.114]	5.494 [2.091-8.511]	0.727 [0.58-0.966]	1.631 [1.054-3.665]	1.58 [0.878-2.551]	1.023 [0.733-2.926]	10.54[5.208-15.807]	0.864 [0.682-2.864]	2.17 [1.494-5.205]	1.534 [0.693-1.841]
Proteinuria, g/g	0.87 [0.48-2.24]	4.8 [3.28-14.3]	2.78 [1.17-6.48]	2.52 [0.96-5.22]	5.03 [1.56-7.44]	1.5 [0.52-5.25]	1.44 [0.31-4.56]	2.9 [2.3-6.8]	2.4 [2.2-3.13]	1.38 [0.42-3.27]	0.09 [0.06-0.12]
Albuminemia, g/L	36 [30.2-39.75]	28 [21-36.25]	26 [23-32]	22 [20-26]	29 [24.8-32]	27 .5 [21.25-33]	29 [25-35.5]	36 [25-39]	29 [22.25-36]	31 [22-39.25]	32.5 [30.75-40.6]

Supplementary Table 2

**SC** **N = 225**	TTP	TMA (TTP excluded)		a-HUS	t-HUS	Pregnancy	Drugs	Infections	Active malignancy	Malignant HT	Transplantation	Auto-immune disease	Other TMA
	n = 14	n = 211	p-value	n = 11	n = 8	n = 95	n = 12	n = 20	n = 22	n = 11	n = 19	n = 6	n = 7
**PLASMIC score**	6 [5.5-6.25]	5 [4-6]	**0.02**	4 [4-5] *	4.5[4-5.75]	6 [5-6]	4.5 [4-5.75] *	4.5 [4-5.75] *	4 [4-5] *	5 [4-5] *	5 [4-5] *	5 [4-6]	5 [5-5]
Components of the PLASMIC score													
Platelets <30 G/L, n [%]	10 [71]	58 [27]	**0.001**	2 [19]	1 [12]	22 [23]	3 [25]	12 [60]	8 [36]	0 [0]	10 [53]	0 [0]	0 [0]
Hemolysis†, n [%]	14 [100]	204 [97]	1	11 [100]	7 [87]	92 [94]	12 [100]	20 [100]	20 [90]	11 [100]	19 [100]	6 [100]	6 [86]
No active neoplasia, n [%]	14 [100]	179 [84]	0.23	9 [19]	8 [100]	95 [100]	11 [92]	14 [85]	0 [0]	11 [100]	17 [89]	6 [100]	7 [100]
No history of transplant, n [%]	12 [88]	179 [84]	1	9 [19]	7 [87]	95 [100]	9 [75]	13 [65]	22 [100]	11 [100]	0 [0]	6 [100]	7 [100]
MCV < 90 per mm^3 n [%]	9 [64]	134 [63]	1	5 [45]	5 [62]	76 [80]	2 [17]	9 [45]	10 [45]	8 [73]	13 [68]	4 [67]	2 [29]
INR < 1.5 n [%]	13 [93]	189 [90]	1	10 [91]	8 [100]	89 [94]	11 [92]	12 [60]	19 [86]	9 [82]	18 [95]	6 [100]	7 [100]
Serum creatinine < 2 mg/dL, n [%]	9 [64]	147 [70]	0.77	0 [0]	2 [25]	87 [92]	7 [58]	13 [65]	15 [68]	1 [11]	14 [74]	2 [33]	6 [86]
**PLASMIC score risk**			**0.004**										
Low-intermediate (≤ 5), n [%]	3 [21]	127 [60]		11 [100]	6 [75]	29 [30]	10 [83]	15 [75]	20 [91]	10 [91]	15 [79]	4 [67]	7 [100]
High (≥ 6), n [%]	11 [79]	84 [39]		0 [0]	2 [25]	66 [70]	2 [17]	5 [25]	2 [9]	1 [9]	4 [21]	2 [33]	0 [0]
**French score**	2 [0.75-2]	1 [1-1]	0.06	0 [0-0] *	0 [0-1] *	1 [1-1]	1 [0-1.75]	1 [1-2]	1 [1-1.25]	0 [0-0] *	1 [1-2]	0.5 [0-1]	1 [1-1]
Components of the French score													
Platelets < 30 G/L, n [%]	10 [71]	58 [27]	**<0.01**	2 [18]	1 [12]	22 [23]	3 [25]	12 [60]	8 [36]	0 [0]	10 [53]	0 [0]	0 [0]
Serum creatinine < 2.273 mg/dL, n [%]	9 [64]	148 [211]	0.76	0 [0]	2 [25]	87 [92]	7 [58]	13 [65]	15 [68]	1 [11]	14 [74]	3 [50]	6 [85]
**French score risk**			**<0.001**										
Low (≤ 1), n [%]	6 [43]	170 [81]		11 [100]	8 [100]	77 [81]	9 [75]	13 [65]	17 [77]	11 [100]	11 [58]	6 [100]	7 [100]
High (= 2), n [%]	8 [57]	41 [19]		0 [0]	0 [0]	18 [19]	3 [25]	7 [35]	5 [23]	0 [0]	8 [42]	0 [0]	0 [0]

Supplementary Table 3

**MSC**<br>**N = 134**	Standard score	Modified score with **adjusted threshold**	p-value (vs standard score)	Modified score with **unadjusted threshold**	p-value (vs standard score)
PLASMIC Score	**≥ 6**	**≥ 7**		**≥ 6**	
Sensitivity	80% [55-100]	70% [42-98]	0.32	80% [55-100]	> 0.9
Specificity	53% [44-62]	82% [76-89]	**< 0.001**	44% [35-52]	**< 0.001**
PPV	12% [4-20]	24% [9-40]	**0.015**	10% [4-17]	**0.018**
NPV	97% [93-100]	97% [94-100]	0.94	96% [92-100]	0.18
LR+	1.7 [1.2-2.5]	3.9 [2.3-6.9]	**< 0.001**	1.4 [1.0-2.0]	**< 0.001**
LR-	0.4 [0.1-1.3]	0.4 [0.1-0.9]	0.94	0.5 [0.1-1.6]	**< 0.001**
French Score	**= 2**	**= 3**		**≥ 2**	
Sensitivity	70% [42-98]	50% [19-81]	0.16	80% [55-100]	0.32
Specificity	84% [77-90]	96% [93-99]	**< 0.001**	63% [54-71]	**< 0.001**
PPV	26% [9-42]	50% [19-81]	**0.046**	15% [5-24]	**0.022**
NPV	97% [94-100]	96% [93-99]	0.28	98% [94-100]	0.77
LR+	4.3 [2.5-7.7]	12.4 [4.3-35.8]	**0.021**	2.2 [1.5-3.2]	**0.001**
LR-	0.4 [0.1-0.9]	0.5 [0.3-1.0]	0.30	0.3 [0.1-1.1]	0.77
**MSC (without pregnancy)**<br>**N = 67**	Standard score	Modified score with **adjusted threshold**	p-value (vs standard score)	Modified score with **unadjusted threshold**	p-value (vs standard score)
PLASMIC Score	**≥ 6**	**≥ 7**		**≥ 6**	
Sensitivity	80% [55-100]	70 % [42-98]	0.32	80% [55-100]	> 0.9
Specificity	88% [79-96]	96% [92-100]	**0.025**	77% [66-88]	**0.014**
PPV	53% [28-78]	78% [51-100]	**0.031**	38% [17-59]	**0.017**
NPV	96% [91-100]	95% [89-100]	0.43	96% [90-100]	0.21
LR+	6.5 [3.0-13.9]	20.0 [4.8-82.6]	0.067	3.5 [2.0-6.2]	**0.016**
LR-	0.23 [0.1-0.8]	0.31 [0.1-0.8]	0.45	0.26 [0.1-0.9]	**0.014**
French Score	**= 2**	**= 3**		**≥ 2**	
Sensitivity	70% [42-98]	50% [19-81]	0.16	80% [55-100]	0.32
Specificity	88% [79-96]	98% [95-100]	**0.014**	70% [58-82]	**0.002**
PPV	50% [24-76]	83% [54-100]	**0.029**	32% [14-50]	**0.027**
NPV	94% [88-100]	92% [85-99]	0.25	95% [89-100]	0.64
LR+	5.7 [2.6-12.7]	28.5 [3.7-219.0]	0.09	2.7 [1.6-4.4]	**0.018**
LR-	0.3 [0.1-0.9]	0.5 [0.3-0.9]	0.28	0.3 [0.1-1]	0.65

In black bold: modified score performs significantly better than standard score.
In red bold: modified score performs significantly worse than standard score.

Supplementary Table 4

**SC**<br>**N = 225**	Standard score<br>with **standard threshold**<br>**(≥ 6)**	Standard score<br>with **adjusted threshold**<br>**(≥ 5)**	p-value
PLASMIC Score			
Sensitivity	79% [57-100]	79% [57-100]	> 0.9
Specificity	60% [54-67]	26% [20-31]	**< 0.001**
PPV	12% [5-18]	6% [3-10]	**< 0.001**
NPV	98% [95-100]	95% [89-100]	0.079
LR+	2.0 [1.4-2.7]	1.1 [0.8-1.4]	**< 0.001**
LR-	0.4 [0.1-1]	0.8 [0.3-2.3]	**< 0.001**

Supplementary Figure 1

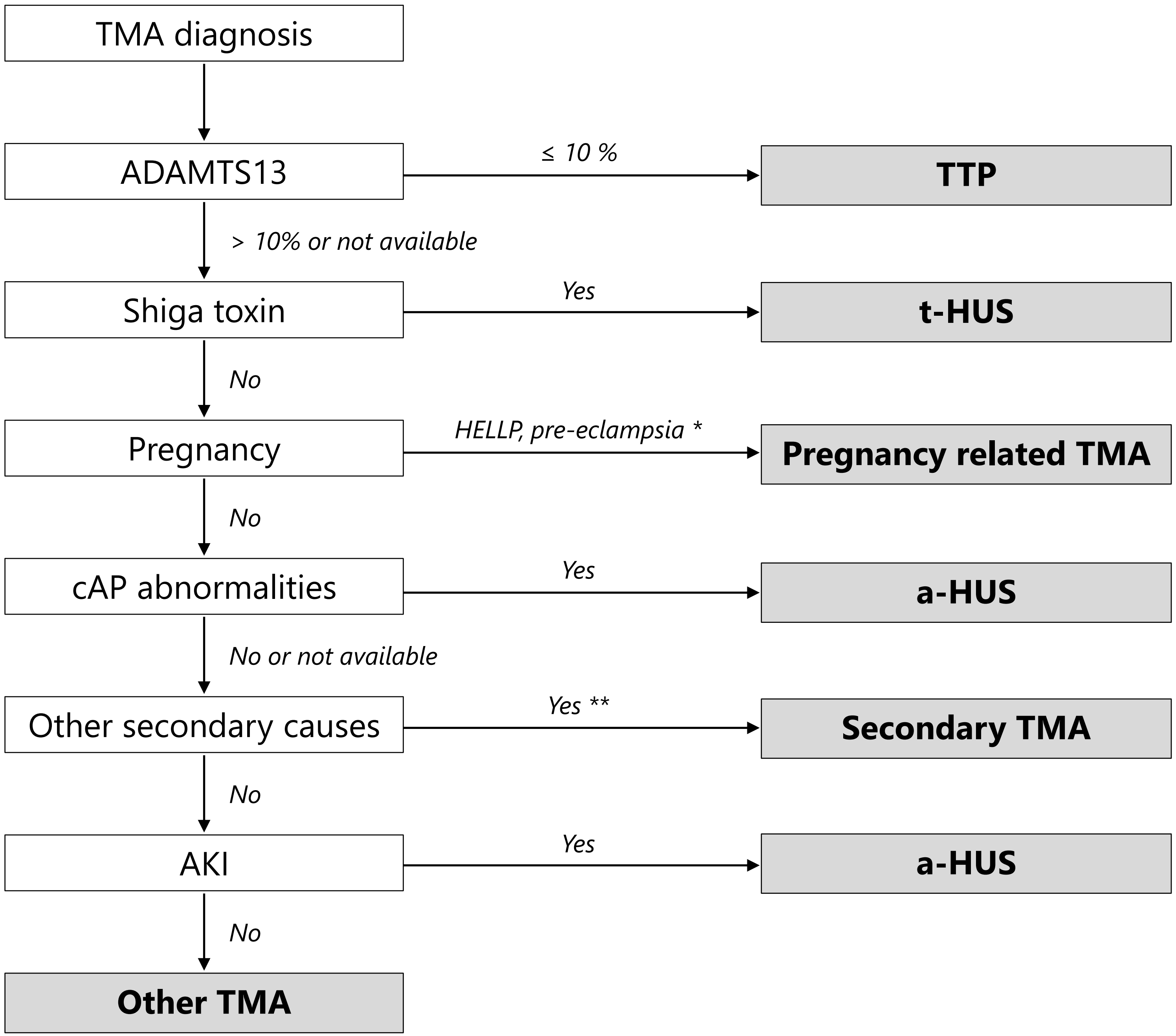

Supplementary Figure 2

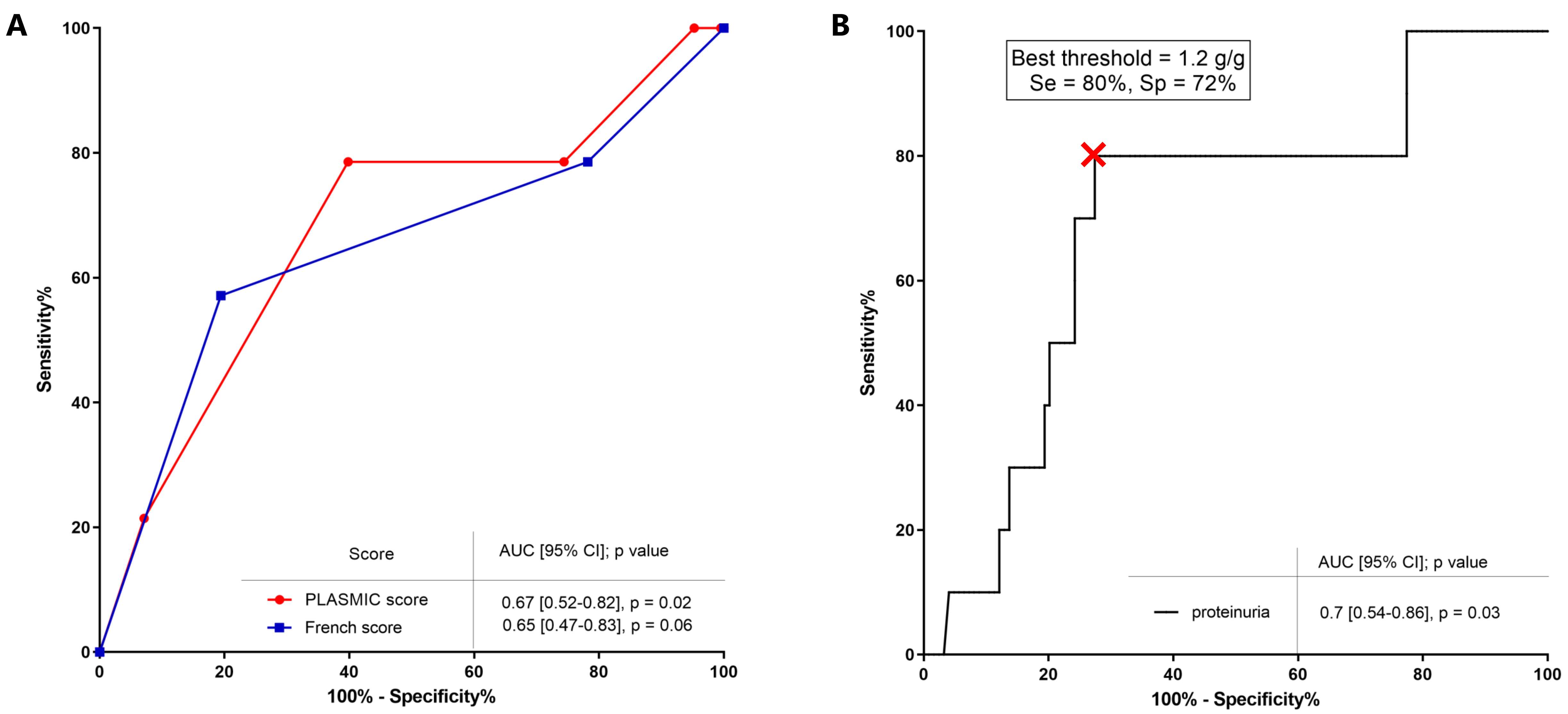

Supplementary Figure 3

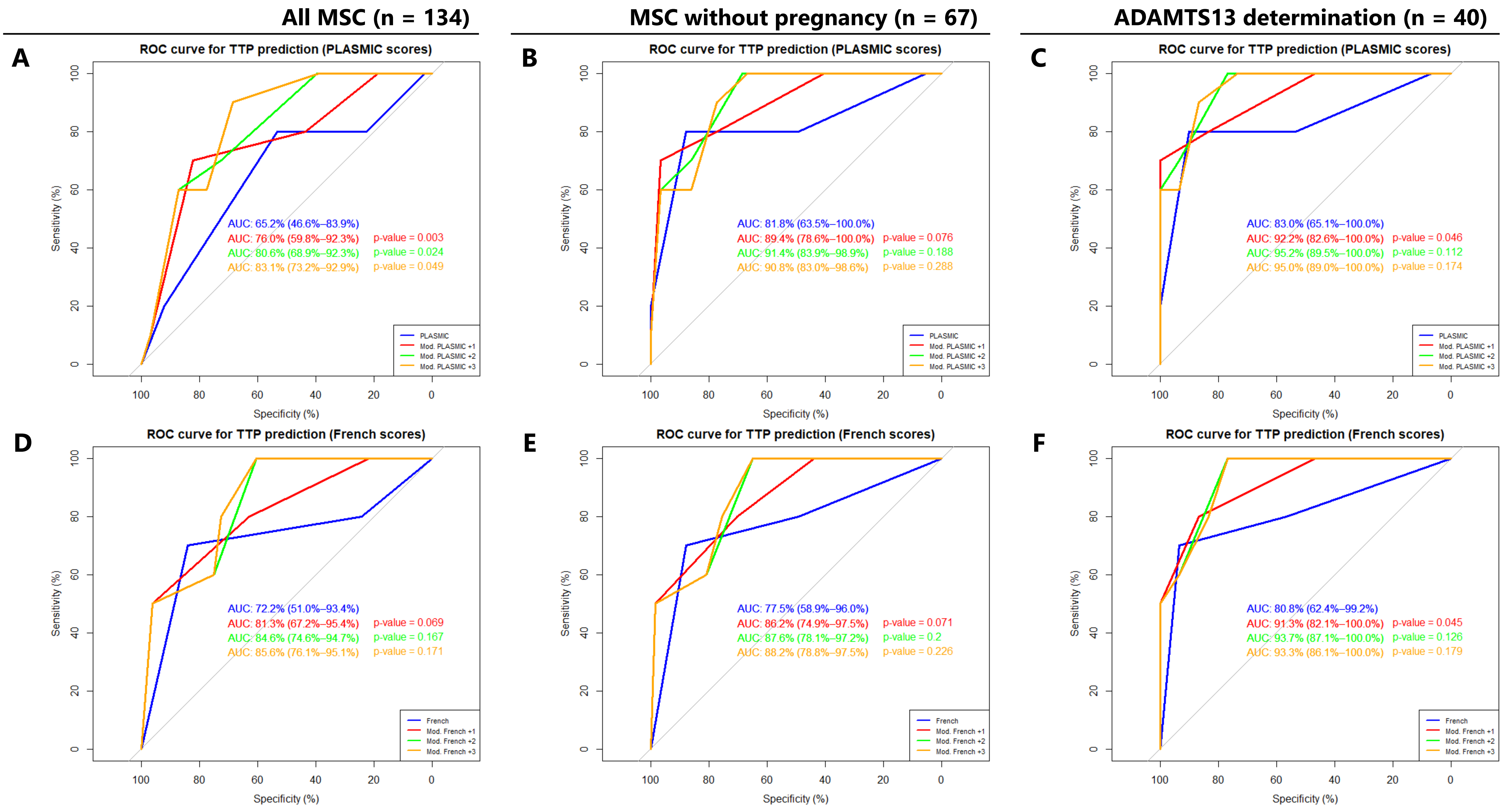

Supplementary Figure 4

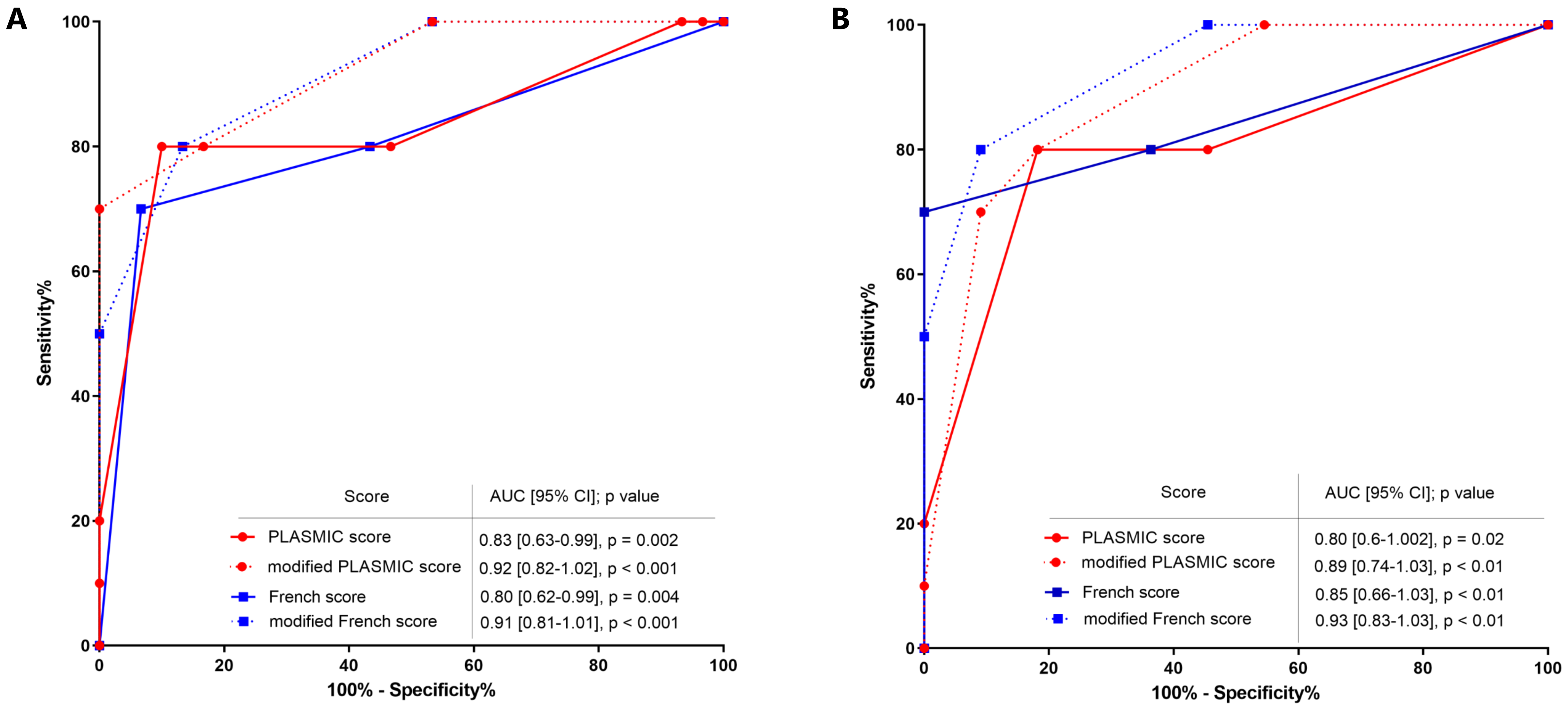

Supplement: Supplementary File (PDF) [file mmc1.pdf]
